# A Pliable Electroporation Patch (ep-Patch) for Efficient Delivery of Nucleic Acid Molecules into Animal Tissues with Irregular Surface Shapes

**DOI:** 10.1038/srep07618

**Published:** 2015-01-05

**Authors:** Zewen Wei, Yuanyu Huang, Deyao Zhao, Zhiyuan Hu, Zhihong Li, Zicai Liang

**Affiliations:** 1 National Center for Nanoscience and Technology, Beijing 100190, PR China; 2 Institute of Molecular Medicine, Peking University, Beijing 100871, PR China; 3 National Key Laboratory of Science and Technology on Micro/Nano Fabrication, Institute of Microelectronics, Peking University, Beijing 100871, PR China

## Abstract

Delivery of nucleic acids into animal tissues by electroporation is an appealing approach for various types of gene therapy, but efficiency of existing methodsis not satisfactory. Here we present the validation of novel electroporation patch (ep-Patch) for efficient delivery of DNA and siRNA into mouse tissues. Using micromachining technology, closely spaced gold electrodes were made on the pliable parylene substrate to form a patch-like electroporation metrics. It enabled large coverage of the target tissues and close surface contact between the tissues and electrodes, thus providing a uniform electric field to deliver nucleic acids into tissues, even beneath intact skin. Using this ep-Patch for efficiently delivery of both DNA and siRNA, non-invasive electroporation of healthy mouse muscle tissue was successfully achieved. Delivery of these nucleic acids was performed to intact tumors with satisfactory results. Silencing of tumor genes using the ep-Patch was also demonstrated on mice. This pliable electroporation patch method constitutes a novel way of *in vivo* delivery of siRNA and DNA to certain tissues or organs to circumvent the disadvantages of existing methodologies for *in vivo* delivery of nucleic acid molecules.

Death of a patient under gene therapy in 1999 led many to dismiss gene therapy as over-hyped and put the field under close scrutiny^1^. However, several clinical successes since 2006, including treatments of patients with the retinal disease Leber's congenital amaurosis^2^,^3^, ADA-SCID^4^, chronic lymphocytic leukemia (CLL)^5^, Parkinson's disease^6^, *etc.*, have bolstered new optimism in the promise of gene therapy. Today, more than 1000 open studies concerning gene therapy were registered on the government database of clinical trial in USA (http://www.clinicaltrials.gov/). Meanwhile, small interfering RNA (siRNA), as a powerful tool for both basic research and clinical application, has attracted continuous attention of investigators all over the world. More than twenty siRNA drugs have entered clinical stage up to now^7^. These siRNAs include drug candidates aiming to cure cancer^8^, HBV^9^, TTR-mediated amyloidosis (ATTR)^10^, diabetic macular edema (DME)^11^, *etc.*

However, difficulties in the delivery of DNA and siRNA *in vivo* have prevented the development of nucleic therapeutics against many diseases^12^,^13^,^14^. Among existing delivery strategies, nucleic acid carriers, both viral vectors and non-viral vectors, have been proved effective for certain clinical applications^7^ albeit concerns about the safety issues of vectors remains^15^. There is however still many tissues and organs that such vectors cannot get efficient access to.

*In vivo* electroporation, as a non-vector-based physical method, has been proved to be capable of delivering DNA into animal tissues^16^. Needle-based electrodes have been widely reported to enhance *in vivo* expression of DNA^17^,^18^. However, the electric field generated by the needle-like electrodes is small and uneven. Since it can only act on a small area of target tissue between each electrode, the needle electrodes have to be sufficiently spaced in order to get a reasonable coverage of tissue surface. This would however require that the electroporation was carried out at high voltage, and such voltages can cause severe damages to the tissue in addition to damage introduced by needle penetration. Largely because of this, the clinical application of needle-based electroporation has been very limited. Recently, micromachined devices were demonstrated for transdermal drug delivery^19^, *in vitro* gene delivery^20^, and *in vivo* bleomycin delivery^21^. The required voltage of electroporation was significantly reduced by introducing micromachined electrodes. However, non-invasive delivery of nucleic acid molecules to tissue beneath the skin by electroporation has not been successfully explored.

Previously, we reported a novel surface electroporation microchip with great performance and compatibility with the standard multi-well plate used in biological research, wherein a novel annular interdigitated electrode design makes it possible to achieve efficient cell transfection as high as 90% under low-strength electrical pulses *in vitro*^22^. However, such a microchip was fabricated on rigid glass substrate and is inappropriate for *in vivo* application since the tissue surface and the chip cannot contact each other closely.

In this work, a micromachined pliable electroporation patch (ep-Patch) utilizing flexible parylene substrate and gold rectangular electrodes was fabricated. Using this ep-Patch, successful delivery of plasmid DNA into healthy muscle tissue from mice was achieved. The electroporation parameters and chip fabrication were optimized and the non-invasive electroporation method was validated by delivering both plasmid DNA and siRNA into muscle and tumor tissue beneath the skin. More significantly, siRNA-mediated suppression of genes in tumor tissues was demonstrated using the described electroporation method on living animals.

## Methods

### Materials

Cy5-labeled siRNA (Cy5-NC) and siLuc (targeting firefly luciferase) were supplied by Suzhou Ribo Life Science Co., Ltd. (Jiangsu, China). Their sequences were as follows: Cy5-NC: sense: 5′-Cy5-CCUUGAGGCAUACUUCAAAdTdT-3′, antisense: 5′-UUUGAAGUAUGCCUCAAGGdTdT-3′; The Cy5 fluorophore was labeled at the 5′ of the sense strand. siLuc: sense: 5′-CCCUAUUCUCCUUCUUCGCdTdT-3′, antisense: 5′-GCGAAGAAGGAGAAUAGGGdTdT-3′. Transfection efficiency of plasmid DNA was determined by using the RFP (pmRFP-C1) plasmid encoding a red fluorescence protein or EGFP (pEGFP-C3) plasmid encoding an enhanced green fluorescent protein. Purifications of plasmid DNA were performed using an EndoFree Plasmid Maxi Kit (Qiagen, German). Optimal cutting temperature (OCT) compound was from Sakura Finetek USA, Inc. (Torracne, CA90501, USA). DAPI (4′, 6-diamidino-2-phenylindole, for staining nuclei) was from Zhongshan Goldenbridge (Beijing, China). Fluorescein isothiocyanate-labeled phalloidin (for staining F actin) and hyaluronidase were supplied by Sigma-Aldrich, USA. Terumo® Insulin Syringe (1 ml·29G × 1/2, 0.33 × 13 mm) was purchased from Terumo Medical Cooperation (Japan). Dulbecco's Modified Eagle's Medium (DMEM), Opti-MEM, L15, fetal bovine serum (FBS), penicillin-streptomycin, and trypsin were purchased from several subsidiary companies of Life Technologies, Inc. (Carlsbad, CA, USA). Cell culture plates and serological pipettes were from Nest Biotechnology Co.,LTD (Wuxi, China). D-Luciferin potassium salt was supplied by Synchem UG & Co. KG (bc219, SynChem OHG, Kalles, Germany). Pentobarbital sodium was provided by Peking University Laboratory Animal Center.

### Finite element analysis (FEA)

A FEA software Comsol V3.5a was used to analyze the electrical field distribution of the ep-Patch. In detail, we utilized the “Electrostatics” model in the MEMS module. The material of the electrodes and the medium surrounding the electrodes were set as “pure gold” and “phosphate balanced solution” respectively. The geometric parameters were set according our design. The solution boundaries were set as electric insulation. A fine triangle mesh method with more than 3 × 10^5^ elements was used to ensure accurate results.

### Cell lines and culture

For *in vitro* electroporation HEK-293 cells were grown in Dulbecco's modified Eagle's medium (DMEM) supplemented with 10% fetal bovine serum, 100 units/ml penicillin, and 100 µg/ml streptomycin. Cells were incubated at 37°C in 5% CO_2_ humidified atmosphere. Breast carcinoma cell line MDA-MB-231 was obtained from the Cell Resource Center of Peking Union Medical College (Beijing, China). MDA-MB-231-Luc that stably expresses firefly luciferase was a gift from Prof. Jun Wang (School of Life Sciences, University of Science and Technology of China) and maintained in our laboratory. For tumor inoculation MDA-MB-231 cells were culture with L15 medium supplemented with 10% fetal bovine serum, 100 units/ml penicillin, and 100 µg/ml streptomycin. To investigate the tumor suppression by siRNA, MDA-MB-231-Luc cells were cultured using the same protocol as MDA-MB-231.

### Animals

Male C57BL/6 mice (for muscle electroporation, weighing 18–22 g) and female BALB/c nude mice (for tumor electroporation, weighing 18–22 g) were purchased from the Academy of Military Medical Sciences of China. Animals were maintained in Peking University Laboratory Animal Center (an AAALAC-accredited and specific pathogen free (SPF) experimental animal facility). All procedures involving experimental animals were performed in accordance with protocols approved by the Institutional Animal Care and Use Committee of Peking University.

### Muscle electroporation in C57BL/6

In the experiment to electroporate the leg muscle non-invasively, the C57BL/6 mice were anesthetized by intraperitoneal (i.p.) injection with pentobarbital sodium (50 mg/kg) and carefully depilated on the thigh using Gillette® Blue Blade (Gillette, Boston, Massachusetts, USA) before intramuscular administration of hyaluronidase. A syringe was penetrated into muscle issue of the mouse thigh with about 3 mm depth beneath the surface, and 40 μl (2 μg/μl) hyaluronidase was injected into several positions of muscle tissue by changing the needle head direction, which covered around 0.8–1.0 cm^2^. After reaction for 30 min, 40 μl RFP plasmid (1 μg/μl) was injected into muscle at the same area using the same method. Twenty minutes later, the muscle of testing mice was covered by the ep-Patch and 5 electric pulses, provided by Electro Square Porator^TM^ ECM 830 (BTX, San Diego, CA, USA), were applied for electroporation. No electric pulses were applied for the control mice. After electroporation, the mice were visually checked for forty-eight hours to determine if there was any skin damage or physical dysfunction.

When optimizing the electroporation parameters in proof-of-concept experiments, we had to assess the *in vivo* transfection efficacy in an invasive way where the mice were anesthetized and shaved, and 0.8–1.0 cm^2^ skin was sheared and isolated from the muscle using operating scissors. Then electroporation was performed according to above-mentioned protocol, followed by stitching the skin and sterilizing the wound. All instruments and materials were sterilized before conducting the experiment.

### Evaluation of RFP expression in mouse muscle tissue

Forty-eight hours (seventy-two hours for invasive electroporation) post electroporation, RFP expression in muscle was examined by *In-Vivo* Imaging System (Carestream In-Vivo Imaging System FX Pro, Carestream Health, USA). The 550 nm excitation and 600 nm emission filters were chosen for the experiment. According to the manufacture's specification, the bandpasses of such excitation and emission filters are 20 nm and 60 nm (wide angle) respectively, suggesting the excitation and emission spectrum are 540–560 nm and 570–630 nm respectively. Other detailed exposure conditions were as follows: Exposure Time: 60.0 sec; X-binning: 2× Binning; Y-binning: 2× Binning; f-Stop: 2.50; FOV: 180 mm; Focal Plane: 13.0 mm. The mice were anesthetized before and during imaging. The RFP signal intensity was quantified by using a molecular imaging software package (Carestream Health, USA). For patch characterization by RFP plasmid expression, presented fluorescent data are the average of two or three independent assays. Each data is showed as the mean ± standard deviation.

### Tumor electroporation in BALB/c nude mice

For tumor electroporation assays, 5 × 10^6^ cells (either MDA-MB-231 cells or MDA-MB-231-Luc cells) were injected subcutaneously into the right axillary fossa of the BALB/c nude mice (female, weighing 18–22 g). The mice were used when the tumor had grown to around 500 mm^3^. For the study of siRNA penetration and distribution in tumor, mice were divided into two groups. Both groups were administered 40 μl (1 μg/μl) Cy5-labeled siRNA intratumorally, but one with electroporation, another without. For study of gene suppression in tumor tissue, mice were randomly divided into five groups (each with five mice) and received the following treatments respectively: group 1, without any treatment; group 2, without siLuc (siRNA targeting firefly luciferase) injection and stimulated by 90 V electrical pulses; group 3, with siLuc injection but without electroporation; group 4, with siLuc injection and electroporation under 70 V; group 5, with siLuc injection and electroporation under 90 V. The detailed electroporation protocol here applied is the same as the above mentioned.

### Tumor frozen section observation

To determine the distribution of Cy5-labeled siRNA in tumor cells, forty-eight hours after electroporation, the tumors were isolated from the animals and placed on Omnisette tissue cassettes, embedded in OCT, and frozen on a foam floater that float on liquid nitrogen contained in a pre-chilled Dewar flask for ~1 min until the OCT turned white and opaque. Subsequently, the specimens were cut into 6 μm sections on a cryostat. Each section was picked up on a glass slide, stained by DAPI for visualize the nucleus and by FITC-labeled phalloidin to visualize F-actin in order to display the cell outline. Finally, cryosections were examined under the confocal microscope (LSM710, Zeiss, Germany). Using image analyze software (ImageJ, NIH, USA), the delivery efficiency was calculated by dividing the number of siRNA-containing cells by the number of all cells.

### Detection of luciferase expression in tumor by *in vivo* bioluminescence imaging

*In vivo* imaging system (Carestream In-Vivo Imaging System FX Pro, Carestream Health, USA) was used to evaluate the firefly luciferase expression level in tumor tissues. Before *in vivo* imaging, animals were anesthetized by i.p. injection of pentobarbital sodium (50 mg/kg) and treated with D-luciferin potassium salt (bc219, SynChem OHG, Kalles, Germany) at the dose of 150 mg/kg of body weight (10 μL/g of body weight, 15 mg/mL stock solution, i.p.) to induce bioluminescence within luciferase-expressing MDA-MB-231-Luc tumor cells. Whole-body images were produced after a standard luminescence exposure starting at ~10 min following injection of D-Luciferin. Detailed exposure conditions were as follows: Exposure Time: 5.0 min; X-binning: 8× Binning; Y-binning: 8× Binning; f-Stop: 2.50; FOV: 180 mm; Focal Plane: 11.0 mm. However, when performing the luminescence detection, the anesthetic degree was various among different mice sincethe individual differences are inevitabe. Accordantly, the times for luciferase substrate D-Luciferin being absorbed into the circulation system, transported to tumor tissues and react with luciferase were slightly discrepant between the different mice. In order to reduce these kinds of errors, we examined the luciferase expression three times independently for each group of mice. In detail, luciferase activity was assessed three times on day 1 and day 2, followed by electroporation of siRNA on day 3. Then its activity was analyzed three times again on day 5 and day 6. The absolute siRNA-treated time frame was 48–72 hours.

Before electroporation, luminescence signal intensity of each mouse at each detection time point was quantified by using software (Carestream Health, USA). The average of three values for each mouse was calculated. Then mean intensity of 4–5 mice of each group was further calculated. Through the same procedures, the mean photon intensity after electroporation for each group was obtained. Finally, for each group the mean value before treatment was used as benchmark (100%), and the value post-treatment was normalized to the benchmark. Quantification data were presented as mean ± SEM.

### Statistical analysis

Data are expressed as the mean ± SD or the mean ± SEM, as indicated in corresponding figure caption. Statistical analyses were performed using Student's *t*-test to measure statistical differences among groups. Data with p<0.05 were considered to be statistically significant.

## Results and discussion

### Design and fabrication of pliable electroporation patch (ep-Patch)

**Figure 1:**
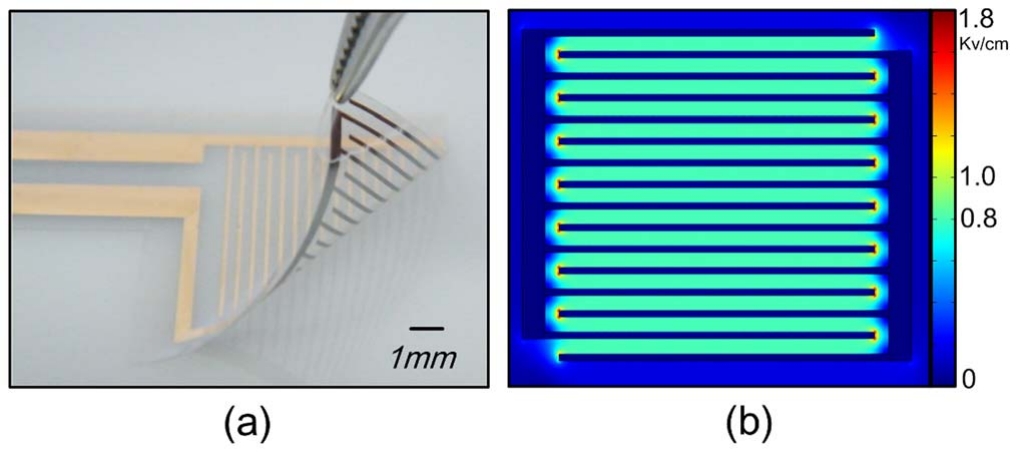
The pliable electroporation patch (ep-Patch). (a) The photo of pliable ep-Patch. The ep-Patch consists of a flexible parylene substrate (10 μm thick) and gold/chrome rectangle parallel electrodes (12 μm thick). The width and spacing of electrodes are 200 μm and 500 μm respectively. (b) The simulated electrical field distribution (by FEA software Comsol Ver.3.5a) of the pliable ep-Patch showed that a 40 V produces an even distributed electrical field with sufficient strength (800 V/cm) for electroporation.

The prototype of the ep-Patch is shown in figure 1(a). A transparent parylene film was used as the substrate for electrodes due to its good flexibility. It enables a tight contact between the ep-Patch and living tissues with different surface profiles (supplementary figure S1). The parylene layer also acts as the insulator between electrodes. Gold was chosen as the material of electrodes because of its good conductivity and biocompatibility. As simulated in figure 1(b), rectangular interdigital microelectrodes were designed to produce a uniform distributed electrical field on the entire ep-Patch area. The width and spacing of the microelectrodes shown in figure 1 were designed to 200 μm and 500 μm respectively. The effective area of the ep-Patch was designed to 100 mm^2^ to fit the typical size of tumor and muscle tissue of experimental mice. By redesigning the layout for lithography, the ep-Patch size can easily be varied according to the needs of different applications.

The pliable ep-Patch was fabricated with MEMS (Micro-electro-mechanical system) processes, including sputtering, lithography, electroplating, and wet etching. The basic fabrication process is shown in supplementary figure S2(a). Using PDS2010 system (Specialty Coating System, USA), a parylene C film was deposited on a 4-inch silicon wafer. A thin gold layer coupled with a chrome adhesion layer was sputtered on the parylene layer, and then the Au/Cr layer was patterned by one mask lithography and wet etched to form electrodes. After being peeled off from the silicon wafer in DI water, the parylene film was diced with proper size to form the proposed ep-Patch. The thickness of gold layer and chrome layer are 0.3 μm and 0.03 μm respectively.

In the basic fabrication procedure described above, the thickness of gold electrode is less than 0.4 μm because of the limitation of the sputtering technology. This is too thin to handle the high current of non-invasive electroporation. To improve the capability of the ep-Patch to handle high current, electroplating was introduced to increase the gold layer thickness to 12 μm. The detailed fabrication process is described in supplementary figure S2(b). Briefly, after sputtering a 0.1 μm gold layer as the seed layer, a 15 μm photoresist (AZ4620, AZ Electronic Materials, Luxembourg) was patterned as the mask for electroplating, and then a 12 μm gold layer was electroplated on the seed layer as electrodes. After removing photoresist and redundant seed layer, the parylene film was peeled off and diced in proper sizes. The difference of metallic luster between sputtered gold layer (thin) and electroplated gold layer (thick) is shown in supplementary figure S2.

### ep-Patch characterizations and *in vitro* nucleic acid transfection

In contrast to previously reported needle-based electroporation devices^23^,^24^,^25^, the ep-Patch was found to have the following prominent features; (i) Flexible parylene substrate (figure 1(a)) has the capability to match different surface profile of targeting tissue. (ii) It can electroporate in a non-invasive way. (iii) It can provide an even electrical field (as shown in figure 1(b)). After covering the tissue with tight contact between ep-Patch and tissue surface (supplementary figure S1), the rectangular interdigital electrodes on the ep-Patch provide an even distributed electrical field to maintain consistent electroporation efficiency on entire ep-Patch area. (iv) The size of electroporation area can be easily adjusted. Finally, (v) It can be easily fabricated by MEMS technology. As most semiconductor products, the cost of a single patch can be significantly reduced by large-scale preparation.

The high efficacy of both plasmid DNA and small interfering RNA (siRNA) delivery of suggested pliable ep-Patch was first verified by *in vitro* cell electroporation (supplementary figure S3). Data manifested that around 80% transfection efficiency was achieved for EGFP plasmid electroporation and treated cells were proliferating well and maintained ideal viability. It was also shown that Cy5-labeled siRNA successfully crossed cell membrane and distributed in cytoplasm, where RNA interference (RNAi) occurred (figure S3(b)).

### Skin-off transfection of DNA into mouse muscle *in vivo*

**Figure 2:**
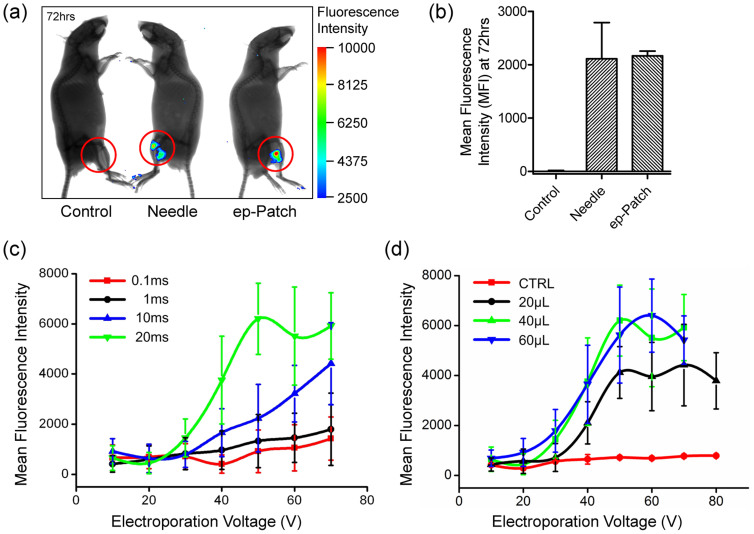
Skin-off transfection of plasmid into mouse muscle and the optimization of electroporation parameters. (a) The whole body fluorescence intensity image of the unelectroporated mouse (left), the mouse treated with needle-based electroporation (middle) and the mouse treated with pliable ep-Patch (right). The fluorescence images were merged with corresponding X-Ray images in order to show the outline of the mice and indicate the physical position where signal comes from. The red circuits label the treatment area. The fluorescent area of right mouse approximately equals the ep-Patch area. Electroporation conditions for the ep-Patch are: 5 electrical pulse; voltage 40 V; 20 ms pulse duration; 2 s pulse interval. Electroporation conditions for electroporation needles are: 5 electrical pulse; voltage 240 V; 20 ms pulse duration; 2 s pulse interval. (b) Quantitative analysis of (a) using a molecular imaging software package (Carestream Health, USA). The mean fluorescence intensity (MFI) was calculated from two exposures of each mouse. Data were normalized to unelectroporated mouse who's MFI was set to zero (background-signal correction). Each bar represents the mean ± S.D. (c) Graphical representation of fluorescence intensity as a function of electroporation voltage, in which the injected volumes of plasmid DNA are 0 (as control), 20 μl, 40 μl, and 60 μl. (d) Graphical representation of fluorescence intensity as a function of electroporation voltage, in which 40 μl plasmid DNA is injected. The electrical pulse durations are 0.1 ms, 1 ms, 10 ms, and 20 ms. In both figure (c) and (d), electroporation voltage is indicated on the horizontal axis, while mean fluorescence intensity of DNA expression is indicated on the vertical axis, and all data are the average of three independent assays. Each data was showed as the mean ± S.D.

Mouse muscle has been widely used for *in vivo* DNA transfection studies for many years. To investigate the capability of the pliable ep-Patch for *in vivo* nucleic acid delivery, we applied RFP plasmid to perform electroporation since the background autofluorescence from mice that comes from food, blood, hair, paws, *ect.* is strong under the EGFP excitation/emission spectrum, and it is therefore hard to distinguish EGFP signal from background signal^26^. Tissue-penetration capacity of near-infrared (NIR) fluorophores are superior to those with shorter wavelength (eg. EGFP)^27^. In the initial proof of concept assays, the skin of mouse leg was incised before electroporation to obtain accurate experimental data. The skin wound was sutured right after electroporation to prevent muscle tissue from infection. All mice were injected with hyaluronidase and RFP plasmid sequentially. By catalyzing the hydrolysis of hyaluronan, a constituent of the extracellular matrix (ECM), hyaluronidase lowers the viscosity of hyaluronan, and thereby increases tissue permeability. It is reported that pre-treatment of the muscle with hyaluronidase could greatly enhance the transfection efficiency of plasmid DNA when used in conjunction with electroporation^28^. The detailed experimental protocol is described in section *Materials and Methods*. The plasmid expressions were observed seventy-two hours after electroporation. As shown in figure 2 (a), the mice without electroporation showed no fluorescence, illustrating no RFP expression in muscle. On the other hand, the mice treated with traditional needle-based electroporation (Supplementary figure S4 for detailed protocol) used as positive control, showed good level of RFP expression. It was observed that that RFP expression area was divided into two separate parts around the two electroporation needles, which proved that electric field was not even for the traditional needle-based electroporation system. The mice electroporated with 40 V electrical pulses with our pliable ep-Patch showed high and even fluorescence intensity, indicating an efficient and even DNA transfection. The fluorescent area and the ep-Patch area were approximately equal, which indicated that the whole ep-Patch fitted tightly with the tissue profile, and the electric field applied on the tissue was even as expected. This was further confirmed by quantitative analysis (figure 2 (b)) of two exposures for all three mice.

### Optimization of *in vivo* electroporation parameters

To optimize the performance of the pliable ep-Patch, some important parameters were determined by quantitative analyzing fluorescence intensity. In previous studies^22^,^29^, we illustrated that the voltage and the pulse duration are two major parameters determining electroporation efficacy. Therefore, the effects of varying pulse duration and voltage were first investigated. As shown in figure 2 (c), under the same voltages, increasing the pulse duration from 0.1 ms to 20 ms resulted in a significant increase in transfection efficiency. When pulse duration was set at 20 ms, the fluorescence intensity first increased and then reached a plateau when further increasing the voltage. The fluorescence intensity reached its peak value when voltage was 50 V. In figure 2(c), it was illustrated that higher voltage and longer pulse duration lead to higher fluorescence intensity. However, prolonged pulse duration and excessive voltage may induce tissue damage, since the high current passing through electrodes probably causes tissue burns, and even worse, causes tissue dysfunctions. With the pliable ep-Patch, mice treated with optimized electrical pulses (20 ms and 40 V) were found to have their physical health well maintained.

We then investigated the effects of nucleic acid amount on fluorescence intensity. As shown in figure 2(d), similar profiles of transfection versus voltage were observed when 20 μl, 40 μl and 60 μl RFP plasmid was injected separately. With 40 µl and 60 µl RFP plasmid we achieved about the same electroporation efficiency, while 20 μl RFP plasmid was with lower fluorescence intensity. Therefore, 40 μl (40 μg) nucleic acid was employed in following assays.

### Non-invasive electrotransfer of DNA into mouse muscle

**Figure 3:**
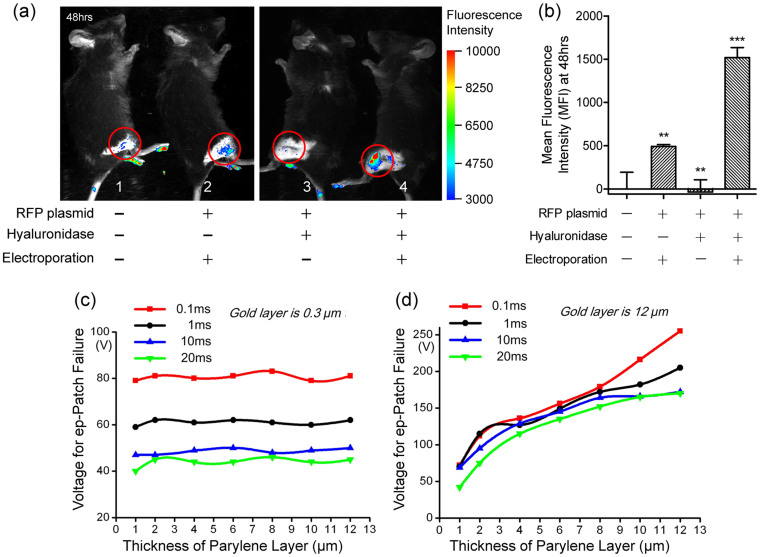
Non-invasive electroporation of plasmid into mouse muscle and fabrication improvement. (a) The whole body fluorescence intensity imaging indicates efficient RFP plasmid expression in electroporated mouse muscle. Mouse leg 1 was without any treatment, which serves as negative control. Mouse leg 2–4 received following treatments respectively: 2: with RFP plasmid injection, electroporation, but without hyaluronidase administration; 3: with RFP plasmid and hyaluronidase injection, but without electroporation; 4: all RFP plasmid, hyaluronidase and electroporation were applied. Fluorescent images were merged with visible-light images to monitor the physical integrity of mouse skin. The red circuits label the treatment area. Electroporation conditions are: 5 electrical pulse; voltage 70 V; 20 ms pulse duration; 2 s pulse interval. (b) Quantitative analysis of (a). The mean fluorescence intensity (MFI) was calculated from 2–3 exposures of each mouse. Data were normalized to unelectroporated mouse who's MFI was set to zero. Each bar represents the mean ± S.D. **P <0.01, and ***P <0.001 vs untreated mouse leg 1. (c) and (d) are the relationships among the thickness of parylene film, the electrical pulse duration and the maximum voltage ep-Patch can withstand when the thicknesses of gold electrodes were 0.3 μm (c) or 12 μm (d).

The invasive operation of needle-based electroporation devices caused inevitably physical wounds, and the intensive electric field around the needles could cause severe electrical burning which increases the risk of infection. Therefore, non-invasive electroporation was desired for *in vivo* study and practical medical application. Using the ep-Patch in a well-controlled study, we have verified that it is feasible to electroporate mouse muscle beneath the intact skin non-invasively (figure 3(a) and (b)). Mouse leg 1 was the negative control without treatment, and leg 3 was the negative control with intramuscular injection of 40 μl (2 μg/μl) hyaluronidase and 40 μl (1 μg/μl) RFP plasmid sequentially, but without electroporation. Mouse leg 2 was injected with 40 μl (1 μg/μl) RFP plasmid, and leg 4 was pre-treated with hyaluronidase prior to injection of 40 μl (1 μg/μl) RFP plasmid. The ep-Patch was subsequently directly attached on the pre-shaved skin, and electrical pulses were used to generate a proper electrical field underneath the skin for electroporation of muscle tissue for these two legs. Forty-eight hours later, both leg 2 and 4 showed obvious fluorescence, indicating efficient expression of RFP plasmid (figure 3 (a)). In addition, leg 4 showed much higher MFI (mean fluorescence intensity) than leg 2, suggesting that hyaluronidase facilitated gene delivery^28^. In contrast, mouse leg 1 and 3, which were without electroporation, showed no fluorescence no matter if RFP plasmid and hyaluronidase were administered or not. These results were further confirmed by quantitative MFIs that were normalized to the untreated mouse leg 1 (figure 3 (b)). For non-invasive electroporation, higher voltage (70 V) was required to obtain a fluorescence intensity similar to what was obtained at 40 V in the assays of skin-off electroporation (figure 2). According a three-dimensional simulation of the electrical field distribution (supplementary figure S5), for tissue within a 1.2 mm distance from the ep-Patch, the electrical field is strong enough for electroporation.

Compared with the needle-based electroporation devices, the ep-Patch utilized non-invasive operation and lower voltage. Theoretically, the ep-Patch would reduce the tissue damage caused by physical invasion and electrical burning. In non-invasive electroporation (70 ~ 90 V), mice were carefully monitored for one week. Neither significant skin lesion nor functional impairment of muscle was observed. To further evaluate the tissue damage, histological examination of the muscle and the skin were performed (supplementary figure S6). Briefly, the histological analysis revealed that the needle-electroporation induced severe damages, including inflammation and physical trauma, on both muscle and skin. Apart from the normal operational voltage (240 V), a much lower voltage (130 V, which was insufficient for electroporation) also induced significant tissue damage. In contrast, the ep-Patch barely caused damage on muscle, with a voltage range from 70 V to 110 V. Slight inflammation was observed when 130 V was applied. For skin, our ep-Patch was proved safe in the operational voltage range (70 ~ 90 V). Obvious skin damages were observed at higher voltage for ep-Patch, however, these damages were found almost recovered on day 7 post treatments.

Apart from the needle-based devices, several tweezers-like and plate-like electrodes have been established for non-invasive electroporation^30^,^31^,^32^. During the non-invasive electroporation process employing these devices, once the anode was placed on an anatomical location of interest, the cathode should be placed on the opposite side of the tissue. Under this circumstance, a strong electric field was applied on the entire tissues between two plates (or tweezers), even though most of these tissues were not supposed to be electroporated. Besides, due to the wild space (more than 1 cm) between two between two electrodes, the EP voltage of either plate-like or tweezers-like devices should be higher than 200 V. Such a high voltage would induce severe tissue damage. By replacing the rigid electrodes with some flexible materials, these tweezers-like or plate-like electrodes could be improved for better tissue adaption. However, the main disadvantages of these kinds of electrode setup (high voltage and unnecessary influences on irrelevant tissues) would remain. In contrast, utilizing micro-patterning allowed us to integrate anode and cathode in the same parylene film. Therefore we could use only one parylene film as electrodes for electroporation, limiting the strong electric field only in the anatomical location of interest, avoiding the negative effects on irrelevant tissues. Also, the spacing between the electrodes could be reduced by micro-patterning according demands, therefore significantly lowering the electroporation voltage to 70 V. While employing two parylene films coated with unpatterned gold layer as electrodes, both 70 V and 90 V was proved insufficient for successful electro-transfection (supplementary figure S7).

### Improvement of ep-Patch fabrication

In non-invasive electroporation assays, the current passing through the electroporation chip could be as high as 600 mA (supplementary figure S8). The high current could fuse the gold electrode. Besides, the accumulating thermal effect could also damage the integrity of parylene film. In our assays, the ep-Patch with thin layer (0.3 μm) of gold electrode could barely handle the current of electroporation. We investigated the thickness of gold layer and parylene layer as influential factors of ep-Patch failure, including electrode fusion and parylene film damage. First, the thickness of gold layer was fixed at 0.3 μm while the thickness of parylene layer was varied from 1 μm to 12 μm. We noticed no significant change in ep-Patch failure over the whole range of thickness of parylene layer. In contrast, as shown in figure 3(c), the critical voltage for ep-Patch failure was reduced from about 80 V to 40 V as the pulse duration increased from 0.1 ms to 20 ms, and the ep-Patch failure occurred on gold electrodes. We further introduced ep-Patches to increase the thickness of gold layer to 12 μm. As shown in figure 3(d), for all four pulse durations, the critical voltage for ep-Patch failure raised from about 50 V to 200 V as the thickness of the gold layer increased from 1 μm to 12 μm. Under these conditions, the gold layer was thick enough to handle the current of electroporation, but the heat accumulation caused by high voltage could still crash the parylene layer. Besides, a voltage higher than 120 V would cause obvious burning of skin. As a good balance of current endurance and difficulties of fabrication, 12 μm gold layer and 10 μm parylene layer were used for the following non-invasive assays.

### RFP plasmid delivery into tumor

**Figure 4:**
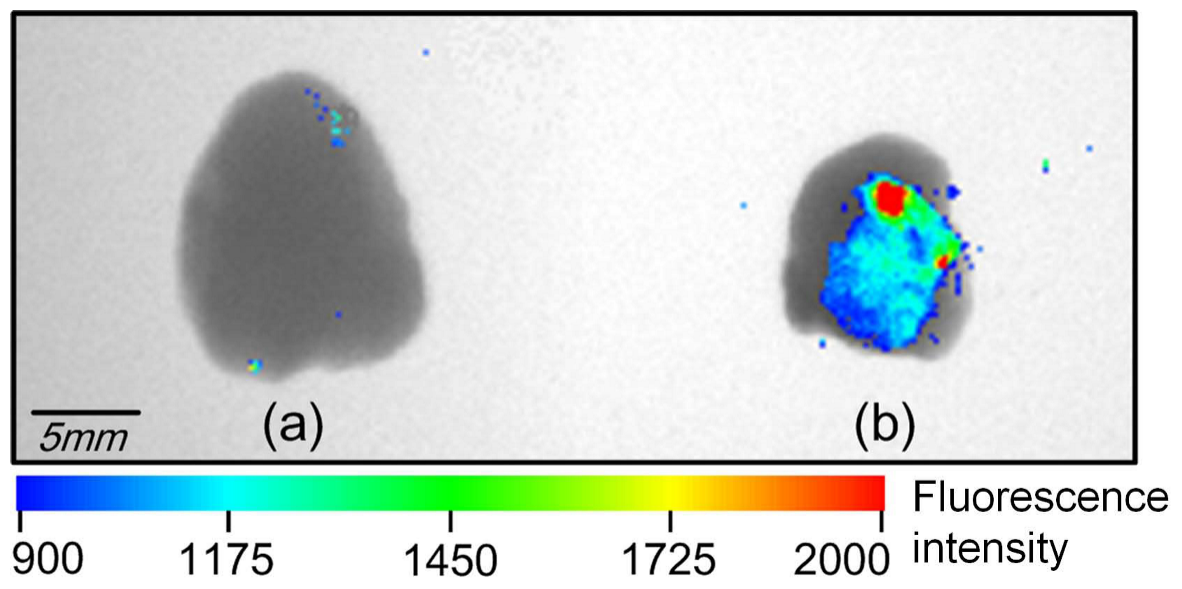
Plasmid DNA transfection in tumor. (a) The tumor with RFP plasmid injection and without electroporation, as negative control, showed no fluorescence signal. (b) The in vivo electroporated tumor tissue showed remarkable fluorescence which indicates successful plasmid DNA expression. Electroporation conditions are: 5 electrical pulse; voltage 70 V; 20 ms pulse duration; 2 s pulse interval.

In order to further assess the capability of mediating *in vivo* nucleic acid delivery and investigate the electrotransfer properties of the ep-Patch, we deployed a xenografted subcutaneous tumor model. In fact, delivery of DNA or siRNA into tumor tissues with high efficiency, low damage, and good uniformity is still a bottleneck for basic research and applications such as gene therapy and drug screening. Compared with muscle, tumors are of much more irregular shapes and traditional needle-based electrodes are inefficient for good coverage of tumor tissues. The same protocol and electrical parameters (70 V, 20 ms pulse duration, 5 pulses with 2 s interval) with muscle electroporation were used to process nucleic acid injection and non-invasive electroporation using RFP expressing plasmid as an indicator. Significant level of RFP expression was observed in tumor tissue treated with electroporation (figure 4(b)), while samples without such treatment showed no RFP expression (figure 4(a)). To our knowledge, this is the first time it has been demonstrated that such ep-Patch can achieve efficient plasmid transportation into tumor cells *in vivo* when the electroporation is performed in a non-invasive manner.

### Electrotransfer of siRNA into tumor tissue and subcellular distribution

**Figure 5:**
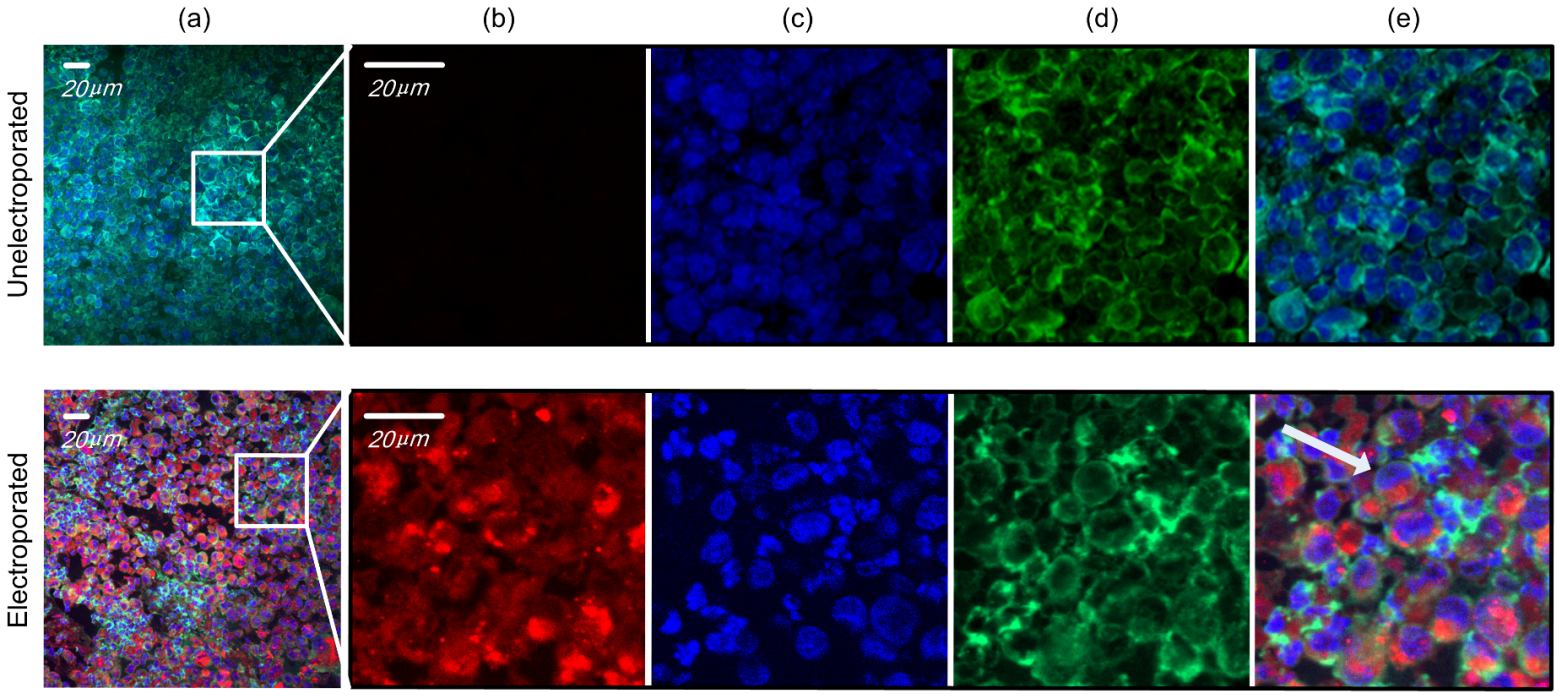
siRNA delivery and subcellular localization in tumor. Confocal laser scanning microscopy (CLSM) images of frozen sections of MDA-MB-231 tumor tissues. Tumor with hyaluronidase and Cy5-labeled siRNA injection but without electroporation showed no fluorescent signal (upper panel). Tumor receiving injection of hyaluronidase and Cy5-labeled siRNA and electroporation exhibited a high electroporation efficiency (>90%) (lower panel). (a) A large merged field illuminated the high efficiency and uniformity. (b, c, d, e) Expanded field from (a); (b) Cy5-labeled siRNAs (red); (c) nuclei stained by DAPI (blue); (d) F-actin stained by FITC-labeled phalloidin (green), to highlight the rough cell outline; (e) merged image of (b), (c) and (d). Arrow showed that the Cy5-labeled siRNA distributed around the nuclei and F-actin staining further confirmed the siRNA stayed in cytoplasm. Delivery efficiency was assessed by dividing the number of all cells with the number of cells containing siRNA. Electroporation conditions are: 5 electrical pulse; voltage 70 V; 20 ms pulse duration; 2 s pulse interval.

We investigated the delivery of Cy5-labeled siRNA into tumor tissue, and the subcellular distribution of such siRNA in tumor cells. Forty microliter hyaluronidase (2 μg/μl) and forty microliter Cy5-labeled siRNA (1 μg/μl) were sequentially injected into the tumor tissues, followed by non-invasive electroporation (figure 5, lower panel) or no further treatment (figure 5, upper panel). Forty-eight hours post electroporation, the tumor tissue was frozen sectioned, stained, and imaged by confocal microscopy. It was found that no fluorescent siRNAs can be observed in tumor cells of mice that were not electroporated, whereas significant levels of siRNAs was take up by cells in mice treated with non-invasive electroporation. In treated cells, the siRNAs was found to be localized in cytoplasm around the nucleus with a high concentration (as white arrow indicates). Image analysis suggested that a large proportion of the tumor cells have been transfected efficiently.

### Suppression of luciferase expression in tumor

**Figure 6:**
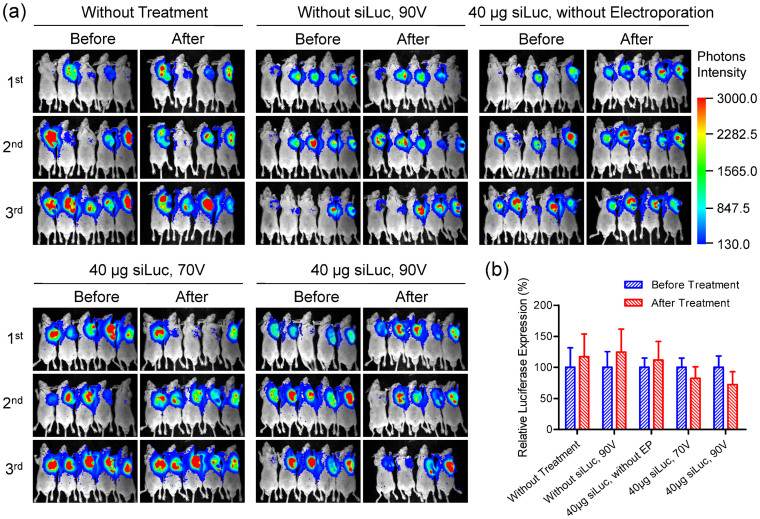
Luciferase suppression in tumor by non-invasive in vivo electroporation. (a) The bioluminescence images of three assays of gene suppression in tumors. The mice were randomly divided into five groups: group 1, without any treatment; group 2, without siLuc (siRNA targeting firefly luciferase) injection, and stimulated by 90 V electrical pulses; group 3, with siLuc injection, but without electroporation; group 4 and 5, with siLuc injection and electroporation under 70 V and 90 V respectively. The lack of one mouse in all three assays of group 1, the third assays of group 3 and 5 was caused by unexpected hyperanesthesia-leaded mice death. (b) The percentage of relative luciferase expression was calculated by normalizing the mean bioluminescence intensities of post-treatment mice to the corresponding mean bioluminescence intensities before treatments. Each bar represents the mean ± SEM. EP: electroporation. The luciferase expression increased by about 20% compared with that before treatment in group 1, 2, and 3. In contrast, luciferase expression were suppressed remarkably (20–30%) in group 4 and 5. Higher inhibition efficiency was observed with increased voltage applied. Collectively, the apparent inhibition efficiency reached up to about 50%.

A subcutaneous MDA-MB-231-Luc (stably expressing firefly luciferase) xenografted murine tumor model was established to validate whether *in vivo* electroporation utilizing the ep-Patch can efficiently mediate siRNA delivery into tumor tissue and further suppress the expression of targeted genes. The bioluminescence intensity in this model is positively correlated with the expression level of luciferase which is stably expressed by MDA-MB-231-Luc tumor cells, so the bioluminescence levels can serve as indicators of the gene expression. When the tumor grew to about 500 mm^3^, the mice were randomly divided into five groups: group 1, without any treatment; group 2, without siLuc (siRNA targeting firefly luciferase) injection, and stimulated by 90 V electrical pulses; group 3, with siLuc injection, but without electroporation; group 4 and 5, with siLuc injection and electroporation under 70 V and 90 V respectively. Then luciferase expression level was examined through detecting the photons produced by the reaction of luciferase and its substrate D-luciferin before electroporation. Forty-eight to seventy-two hours later, luciferase expression level was assessed again for each mouse and compared with the same mouse before the treatment. In order to reduce the errors resulting from individual difference in the mice as much as possible, we measured luciferase expression three times independently both before and after electroporation. Then the average of the three values of photon intensity was calculated for each mouse and the relative luciferase expression level before and after treatment is shown in figure 6. The luciferase activities post-treatment increased by 17% and 24% for group 1 and 2 respectively compared with those before treatment. Since the luciferase gene was stably integrated into the genome of MDA-MB-231 cell, its expression in every single cell should remain at a relative stable level if no stress was given. This suggested that proliferation of tumor cells should have resulted in an increment of luciferase expression by about 20% in this time frame. Data from group 3, where the mice were administered with 40 μg siLuc but without electroporation, a luciferase increment of 12% indicated minimal differences from group 1 and 2. In contrast, luciferase expression post-electroporation of those mice being patch-electroporated were down-regulated with 18% and 28% inhibition efficiency compared with the luciferase levels before electroporation treatment for group 4 and 5 respectively. This result demonstrated that the anti-luciferase siRNAs have totally erased the any increase of luciferase expression due to the proliferation of tumor cells and further lowered the existing luciferase levels. These two groups represented an apparent inhibition efficiency of 30–52% when compared to groups without treatment. It was also found that higher inhibition efficiency was observed as the voltage applied to the mice increased from 70 V to 90 V.

## Conclusion

Electroporation has been demonstrated to be a viable method for delivery of macromolecules such as DNA and siRNA into living animals, but the low efficiency, invasive manner of treatment, and high operational voltage have limited the application of this useful technology in many potential applications. In this paper, a parylene-based electroporation patch (ep-Patch) for *in vivo* nucleic acid delivery has been engineered. Such ep-Patch integrated the advantages of micromachined interdigital electrodes, which created a very even electric field, and the flexibility of the parylene film offered tight surface contact between ep-Patch and targeted tissue. In our experiments, by using this ep-Patch, mouse muscle cells and subcutaneous MDA-MB-231 tumor cells were successfully transfected *in vivo* with plasmid DNA. Non-invasive electroporation of siRNA and DNA over a large area of both healthy tissue and tumor tissue was demonstrated. All electroporated mice maintained good health state and showed no visual damage or functional disability either globally or in the area contacting the ep-Patch. We also provided specific evidence that silencing of a gene expressed within the tumor can be achieved using non-invasive electroporation mediated by our ep-Patch.

Compared with previously reported needle-like, tweezers-like or plate-like electrodes, the ep-Patch was found to have the following prominent features: i) By appropriately arranging the micro-electrodes and closely covering the tissue surface, the ep-Patch focused the electrical field on the targeted tissue, avoiding the unnecessary influence or safety risk on irrelevant tissues; ii) Benefited from the non-invasive operation and the low electroporation voltage, the ep-Patch significantly reduced the tissue damage; iii) The simple fabrication process enabled us to enlarge the effective surface for a bigger transfection area, barely increasing the cost. Meanwhile, once we maintained a certain distance between the electrodes, the voltage would stay low while enlarging the ep-Patch. Therefore, the ep-Patch opens new opportunities for the further improvement of the transfection efficiency and tissue damage in several clinical applications, such as intramuscular DNA vaccination and the electroporation for the internal fragile organs with irregular profile.

## Supplementary Material

Supplementary Information Supplementary Materials
