# Supplementary material for: A Pliable Electroporation Patch (ep-Patch) for Efficient Delivery of Nucleic Acid Molecules into Animal Tissues with Irregular Surface Shapes
Source: Sci Rep. 2015 Jan 5;5:7618. doi: 10.1038/srep07618 (PMC5154598; doi:10.1038/srep07618)
Supplement: Supplementary Information — Supplementary Materials [file srep07618-s1.doc]

Supplementary Material

A Pliable Electroporation Patch (ep-Patch) for Efficient Delivery of Nucleic Acid Molecules into Animal Tissues with Irregular Surface Shapes

Zewen Wei^1*^, Yuanyu Huang^2*^, Deyao Zhao^2^, Zhiyuan Hu^1^, Zhihong Li^3^, Zicai Liang^2^

^1^ National Center for Nanoscience and Technology, Beijing 100190, PR China

^2^ Institute of Molecular Medicine, Peking University, Beijing 100871, PR China

^3^ National Key Laboratory of Science and Technology on Micro/Nano Fabrication, Institute of Microelectronics, Peking University, Beijing 100871, PR China

^*^ These authors contributed equally to the work.

Correspondence should be addressed to Z. Liang ([*liangz@pku.edu.cn*](mailto:liangz@pku.edu.cn), Tel./fax: +86-10-62769862), Z. Li ([*zhhli@ime.pku.edu.cn*](mailto:zhhli@ime.pku.edu.cn), Tel./fax: +86-10-62751789) or Z. Hu ([*huzy@nanoctr.cn*](mailto:huzy@nanoctr.cn), Tel./fax: +86-10-82545643)

**Supplementary Figure S1**

**Photos of proposed ep-Patch, xenografted subcutaneous tumor and scheme of the flexibility of the patch**


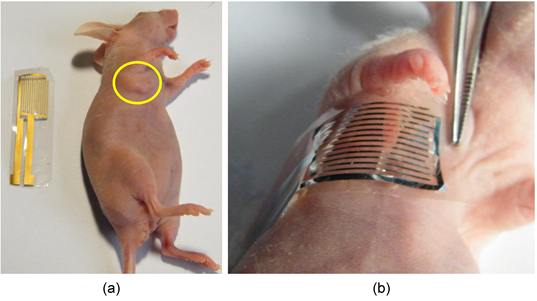


1. The proposed ep-Patch in this study and MDA-MB-231 (or MDA-MB-231-Luc) xenografted murine tumor model. Yellow ellipse indicates the tumor beneath the mouse skin.
2. Benefited from the flexibility of the parylene film, the ep-Patch can easily be bent to fit the irregular profile of tissue. Specifically, while applying the ep-Patch, we used two tweezers to hold both ends of the ep-Patch and kept compressing the ep-Patch on the surface of the targeted tissue, till there were no gap between them. Furthermore, the “tight” contact was indirectly proved by the skin burns. While we perform the skin damage assays, we found that the patch caused regular and paralleled burn lines under a high voltage (130 V). The shape of this imprinting was almost a copy of the profile of gold electrodes array, which demonstrated ep-Patch could touch the skin very tightly and precisely.

**Supplementary Figure S2**

**Fabrication process of pliable electroporation patch (ep-Patch)**


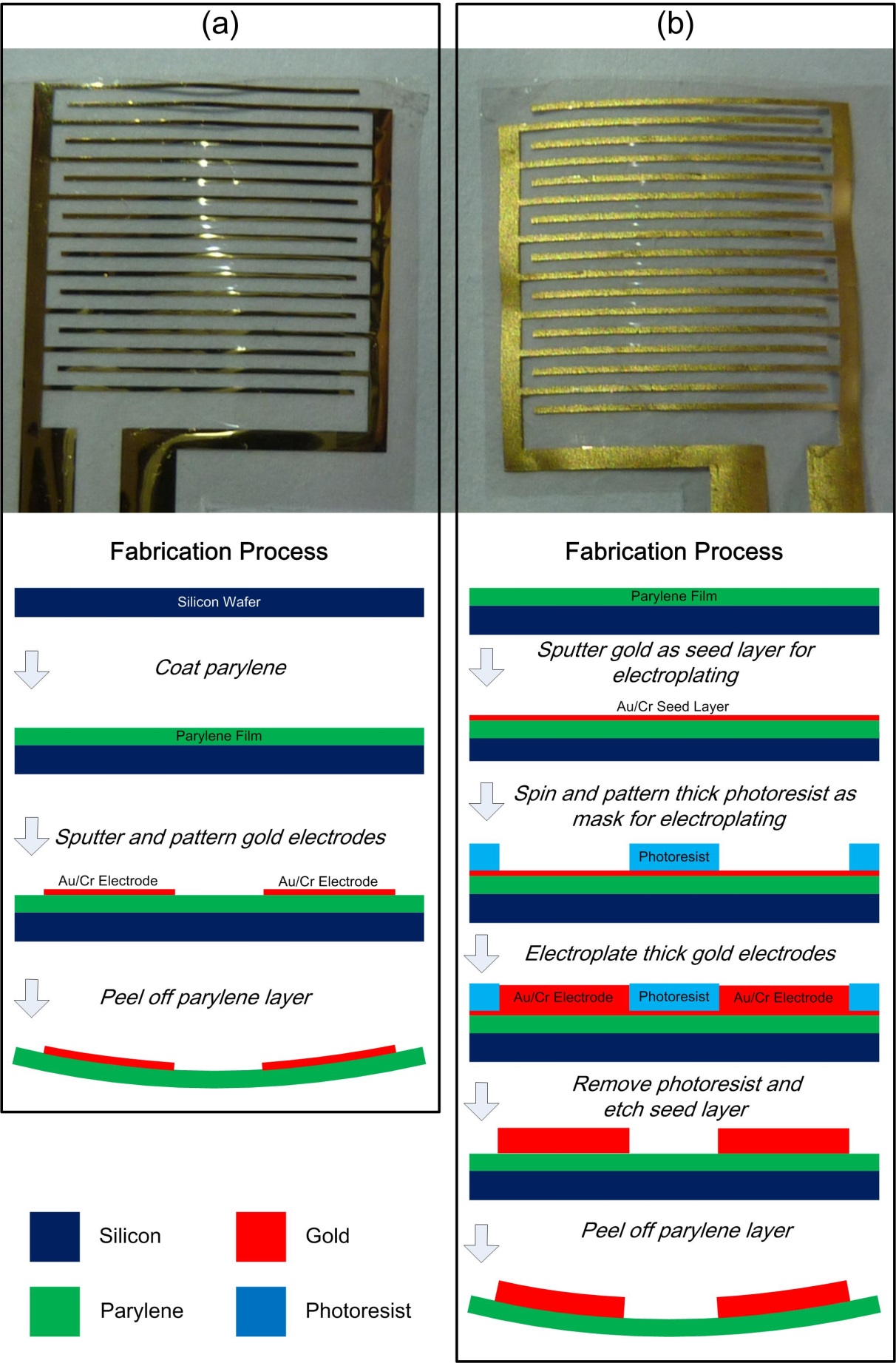


(a) The pliable electroporation patch (ep-Patch) with thin gold electrodes and its fabrication processes.

The pliable ep-Patch was fabricated with standard MEMS (Micro-electro-mechanical system) processes. The fabrication process was started with coating a 10 μm thick parylene C film (using PDS2010 system, Specialty Coating System, USA), as substrate, on a 4 inch silicon wafer. A 0.03 μm chrome layer was firstly sputtered on parylene layer, as adhesion layer between parylene and gold. Then a 0.3 μm gold layer was sputtered on the chrome layer. UV Photolithography was used to pattern a 2 μm thick RZJ304 photoresist (Ruihong Fine Chemistry, China) to the mask for wet etch of gold layer. Then the gold layer and chrome layer was wet etched with potassium iodide solution and ammonium ceric nitrate solution respectively to form electrode. After removing the residual photoresist by nitrosonitric acid, the parylene layer was gently peeled off and diced with proper size. In our experiment, the peeling process was finished in DI water to reduce the possibility of patch crash.

(b) The electroplated 12 μm thick gold electrodes showed different metallic luster from the sputtered thin gold electrodes.

The fabrication process was also started with coating 10 μm thick parylene C film on 4 inch silicon wafer. A chrome adherent layer was employed too. Instead of using sputtered gold layer as electrodes directly, the 0.1 μm sputtered gold layer was used as seed layer for electroplating another thick gold layer. A 15μm thick photoresist (AZ4620, AZ Electronic Materials, Luxembourg) is patterned as mask for electroplating, then 12 μm thick gold layer is electroplated on seed layer as electrode. Hexamethyldisilazane was employed between photoresist and seed layer to enhance the adhesion, therefore avoid the shedding of photoresist during electroplating process. Nitrosonitric acid and oxygen plasma were successively used to thoroughly remove the thick photoresist. Then the whole patch was treated with potassium iodide solution and ammonium ceric nitrate solution respectively to remove redundant Au/Cr seed layer. Finally, the parylene layer was peel off and diced with proper size.

**Supplementary Figure S3**

***In vitro* electroporation of plasmid DNA and siRNA in HEK-293 cells by ep-Patch**


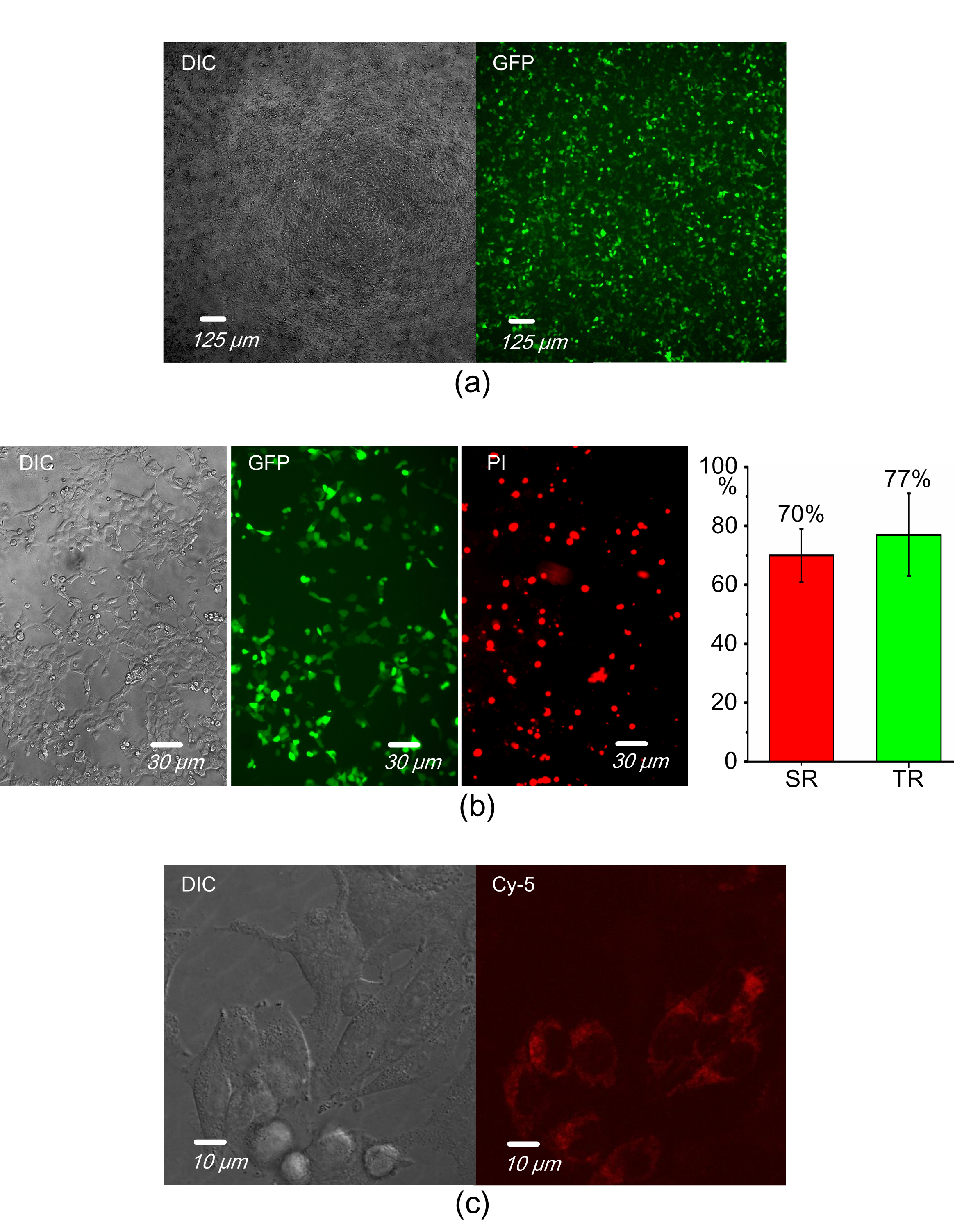


1. Efficient and uniform expression of plasmid DNA by ep-Patch:

Cell line HEK-293 (Human Embryonic Kidney 293 Cells) and green fluorescent protein (pEGFP-C3) were used to characterize the efficacy of *in vitro* cell electroporation. Cells were cultured in a 75 cm^2^ flask for 24 hours, and then were harvested and re-suspended in electroporation buffer [^1^](#_ENREF_1) (there was no buffer used for *in vivo* electroporation) After pEGFP-C3 had been added, 20 μl mixture was dispensed on to patch surface and three electrical pulses (60 V, 0.1 ms, 2 s interval) were applied right away to perform electroporation. Then the mixture was transferred to a 96-well plate filled with enough cell culture medium. As shown in Figure (a), twenty-four hours after electroporation, most of cells expressed the GFP. This reveals that the ep-Patch produced an evenly distributed electrical field

1. Efficiency and cell viability of ep-Patch mediated DNA expression:

To further analyze the transfection efficiency and the cell viability, the number of GFP-expressing cells was counted at five randomly chosen regions; the number of living cells was determined by PI (propidium iodide) exclusion. For each transfection, transfection rate (TR) was calculated by dividing the number of GFP expressing cells by the number of living cells, while the survival rate (SR) was obtained by comparing the number of living cells between treated and untreated samples. Presented data are the average of three independent assays. Each bar represents the mean ± S.D.

1. *In situ* electroporation of siRNA and monitoring of adherent cells:

Cy5-labeled siRNA (red fluorescent area) was used to verify the siRNA delivery by the proposed ep-Patch. To examine if siRNA localized in cell or just attached on the cell membrane, confocal microscopy was introduced to image the cells. Compared with previously reported *in vitro* electroporation devices [^2^](#_ENREF_2), the substrate (10 μm parylene film) of this ep-Patch was thin enough to meet the requirement of confocal microscopy, even with a glass slide covered. To process *in situ* electroporation, cells were firstly cultured on the patch surface for twenty-four hours. Then the culture medium was carefully replaced by electroporation buffer [^1^](#_ENREF_1). After applying electrical pulses for cell electroporation, the electroporation buffer was removed and completed DMEM was added gently to ensure the tight adherent between cells and patch surface. The cells were cultured for twenty-four hours before observed with confocal microscopy. Figure (c) showed that Cy5-labeled siRNA successfully crossed cell membrane and localized in cytoplasm, where RNA interference (RNAi) occurred.

**Supplementary Figure S4**

**The needle-based electroporation system and experimental procedure.**


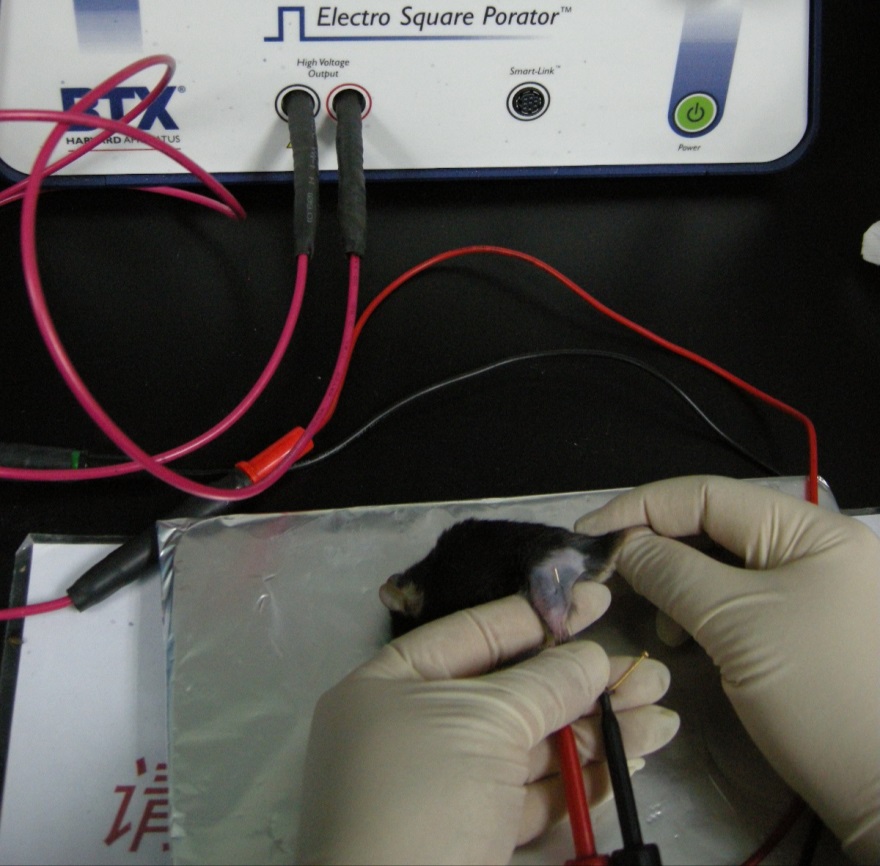


To electroporate mouse muscles using needle electrodes, the mouse was firstly anesthetized, injected with hyaluronidase and RFP plasmid sequentially. The reagents, injection method and reaction time were the same with the protocol used in the patch-based electroporation (described in section of *Materials and methods*). Twenty minutes after RFP plasmid injection, two identical needles were inserted into mouse muscle. The diameter and the length of the needle are 0.8mm and 7 cm respectively. Since it’s difficult to maintain two needles were parallelly placed by manually operation, typical spacing between two needles was 0.6 ~ 0.8 cm. Then 5 electrical pulses, provided by Electro Square Porator^TM^ ECM 830 (BTX, San Diego, CA, USA), were applied for electroporation. The pulse conditions were: voltage 240 V; 20 ms pulse duration; 2 s pulse interval. After electroporation, the needles were extracted and the mouse was fed for 72 hours before fluorescence detection.

**Supplementary Figure S5**

**The simulation of the effective tissue depth of the ep-Patch electroporation**

**
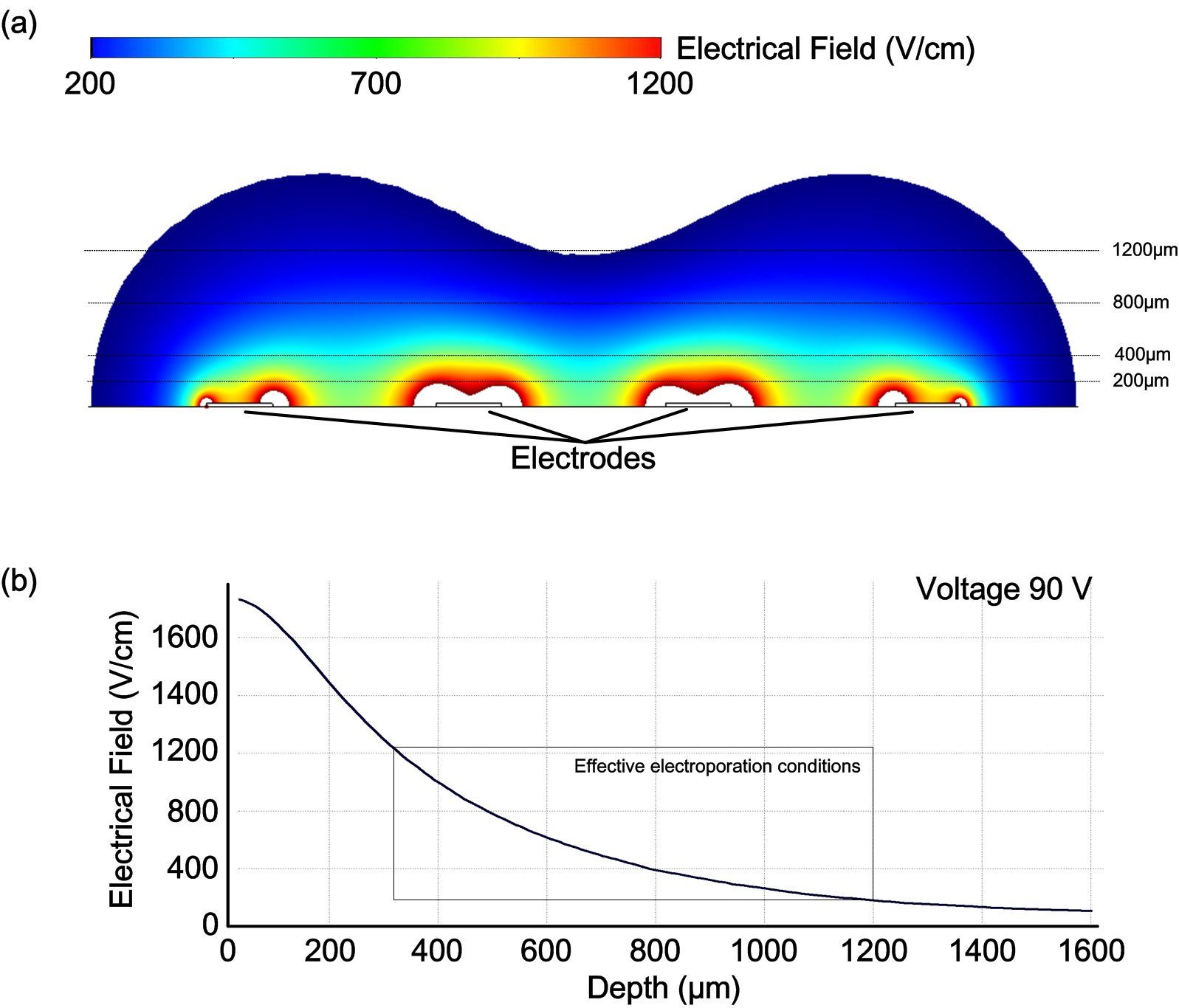
**

(a) As mentioned in the *Materials and methods* section, a FEA software Comsol V3.5a was used to analyze the electrical field distribution of the ep-Patch. A three-dimensional model, including two pairs of gold electrodes and dielectric medium around the electrodes, was built up to analyze the electrical field strength. Previously studies agreed that 200 ~ 1200 V/cm was a proper range of the electrical field for electroporation. Tissue would be damaged under an electrical filed stronger than 1200 V/cm, or remain unelectroporated for an electrical filed weaker than 200 V/cm. Therefore, 200 and 1200 V/cm were set as the higher and lower cut-off value for the chromatic exhibition in figure (a), respectively. Thus the area that the ep-Patch can efficiently affect was indicated by the colored area. The depth the efficient electrical field could reach was about 1.2 mm.

(b) The relationship between the electrical field strength and the depth (along the vertical direction in figure (a)). The effective electroporation conditions were marked by a rectangle, which also indicate the effective depth could reach about 1.2 mm

**Supplementary Figure S6**

**Tissue damage introduced by needle-based electroporation and ep-Patch**


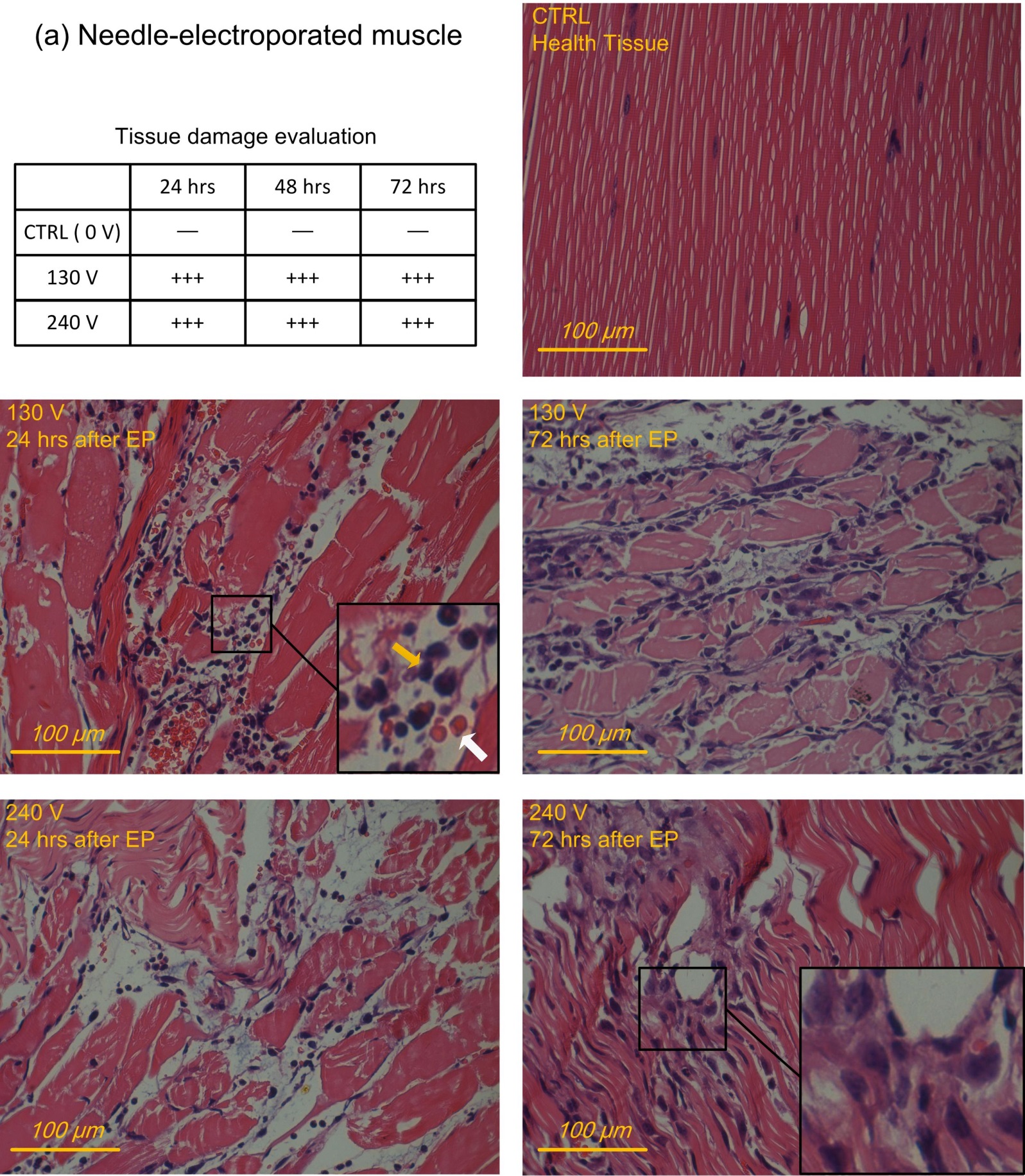


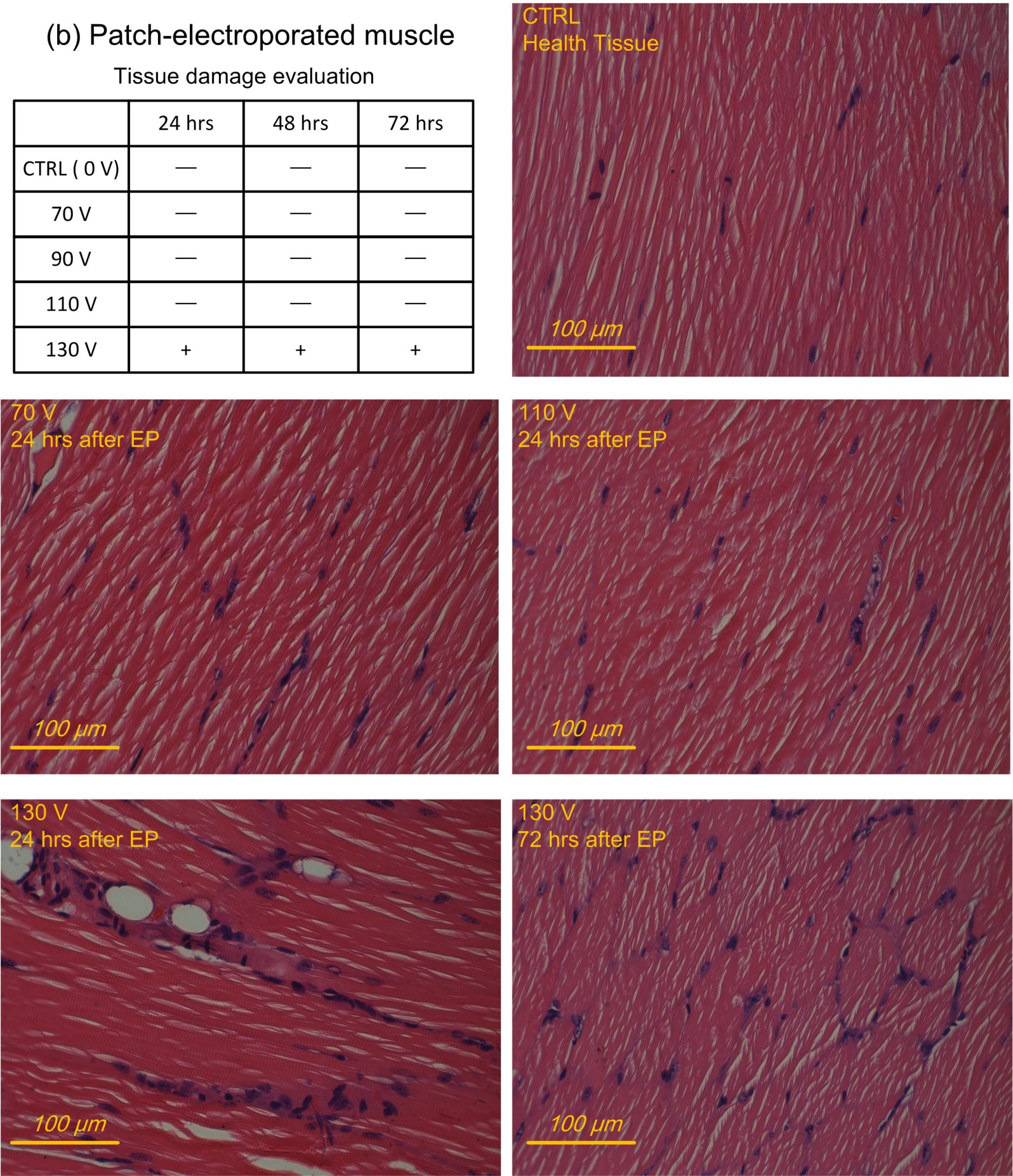


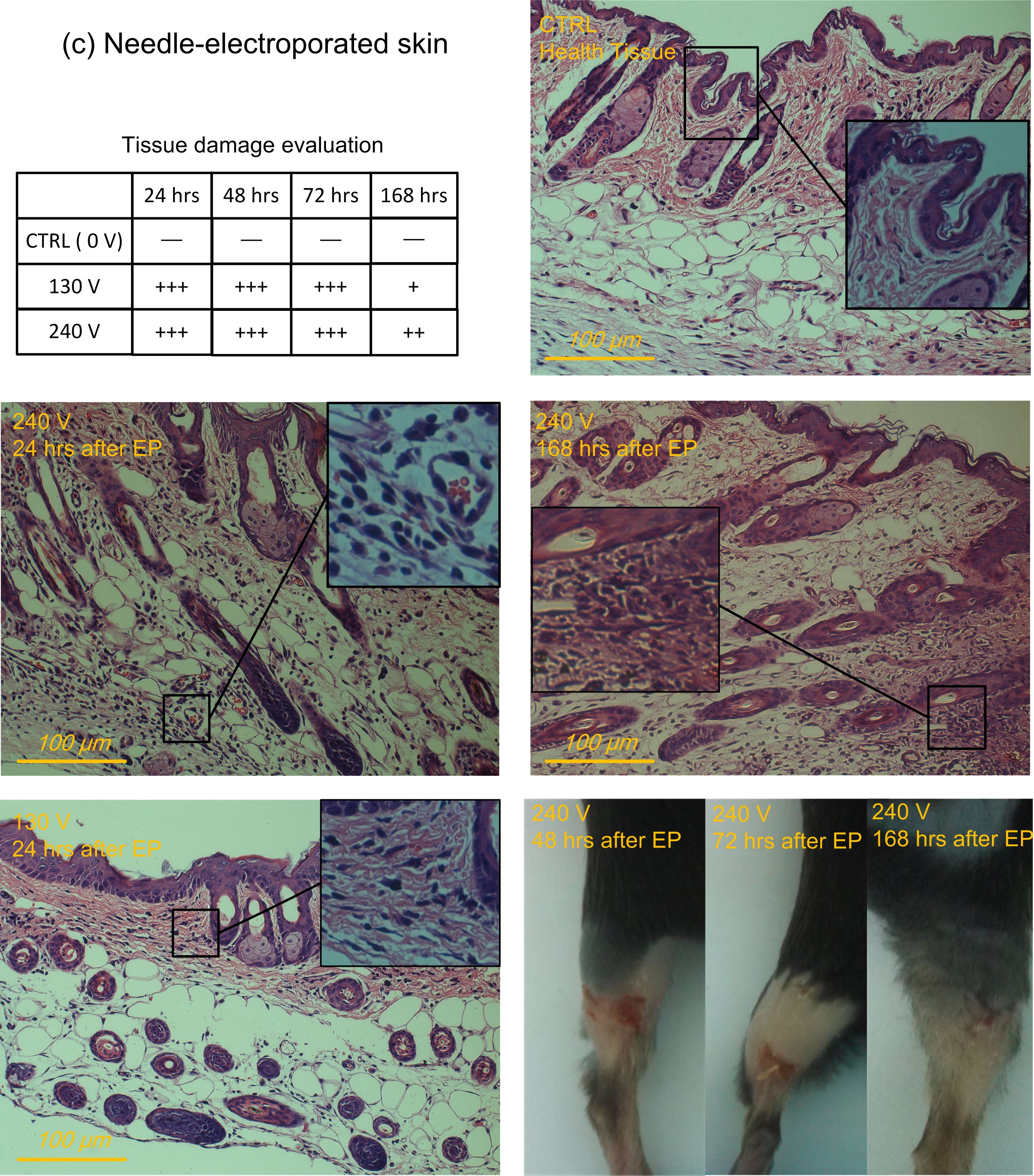


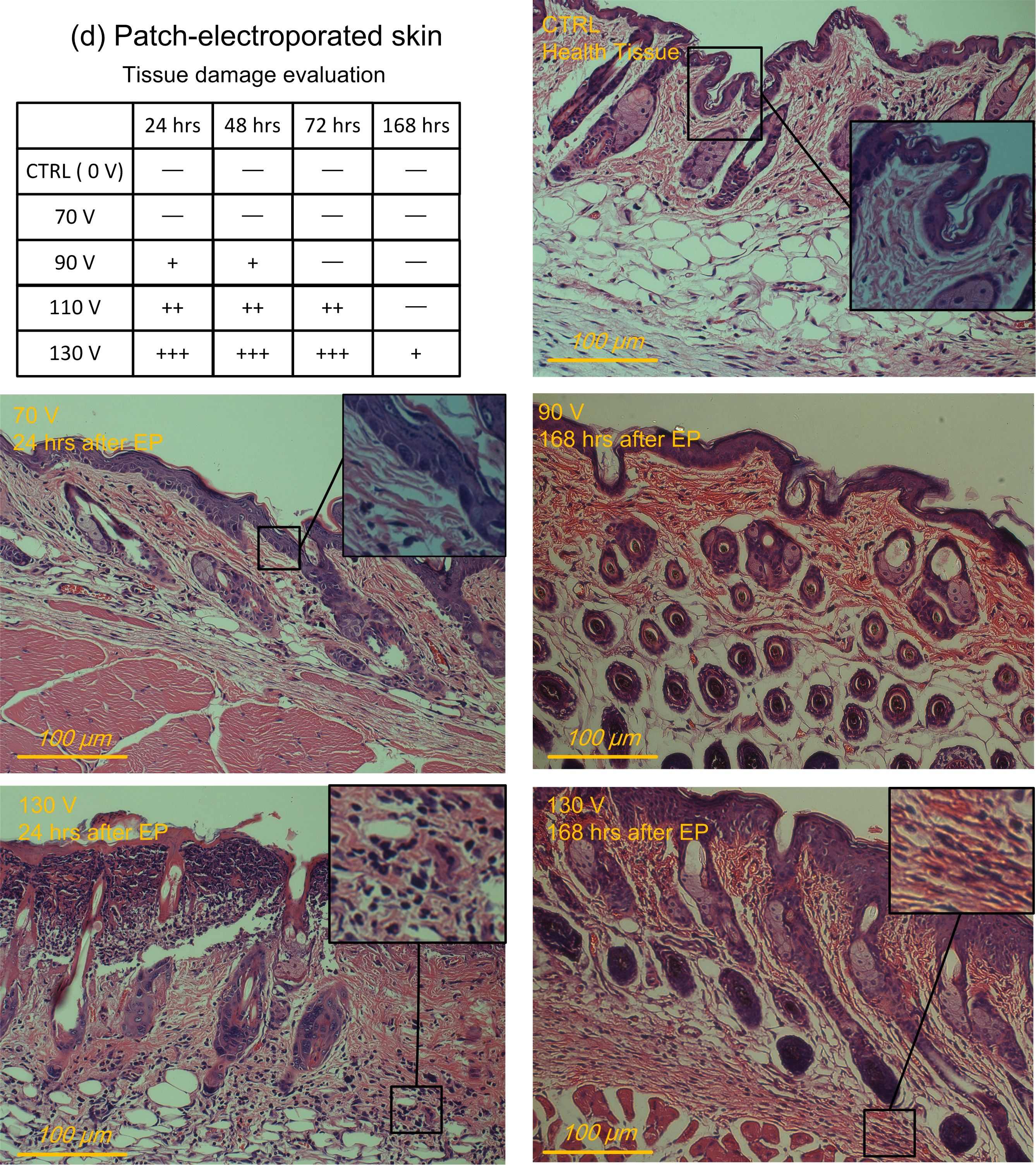


In order to evaluate the damage resulting from electroporation using needle-based device and ep-Patch, further histopathology analysis was performed on normal C57BL/6 mice. Briefly, both two hind legs were depilated using Hair Removal Cream (Veet®, Reckitt Benckiser, UK) 24 hours before electroporation. Mice were randomly divided into seven groups (eight animals per group): group 1, without any treatment; group 2 and 3, electroporation with needles at 130 V and 240 V respectively; group 4-7, electroporation with ep-Patch at 70 V, 90 V, 110 V and 130 V, respectively. Both hind legs of each mouse were electroporated for group 2-7. Two mice of each group were sacrificed by cervical dislocation at 24 hours, 48 hours, 72 hours and 168 hours (7 days) post treatment. Skins and muscles of four legs were collected, followed by fixing in 10% formalin for 24 hours at 37 °C, paraffin embedding, sectioning and staining with H&E. At last, sections were recorded with optical microscope (Olympus X71, Olympus, Tokyo, Japan) to analyze histological change.

To illustrate the damage severity clearer and directly, we introduced a scoring system when analyzing the H&E stained sections. Here, “-” means no damage was observed, “+” means slight damage was found, and “+++” means severe damage was recorded. The damage degree was judged according to the pathological changes, such as inflammation, lymphocytic infiltration, blood cell exudation, physical trauma, *etc.* The damage degree of muscles electroporated with needles at 130 V and collected at 24 hour post treatment was defined as “+++”. The more “+” was given, the severer damage was introduced. Consequently, semi quantitative summary was made and shown.

We firstly investigated the damage on muscle. As shown in figure (a), as expected, needle-based electroporation resulted in severe damage, including significant physical trauma, massive lymphocytic infiltration (indicated with the yellow arrow), and even widely exudation of red blood cells (marked with the white arrow). All above phenomena revealed the needle penetration and electrical-burning due to the high voltage would induce remarkable physical damage, along with the widely-spread inflammation. Even reducing the voltage from 240 V (the normal operational voltage) to 130 V (which was insufficient for electroporation) would barely alleviate the muscle damages. Furthermore, no tissue recovery was observed within 72 hours. In contrast, as shown in figure (b), the ep-Patch caused neither physical trauma nor lymphocytic infiltration with the voltage ranging from 70~110V. Our operational voltage for non-invasive electroporation was from 70 V to 90 V. We further enhanced the voltage to 130 V to explore the safety limit. Sporadic inflammations were found, hinting slight tissue damage was introduced under this condition. However, it was a transient incident since it recovered within 72 hours.

We then analyzed the skin damage. As shown in figure (c), needle-based electroporation also caused significant damage with the voltages from 130 V to 240 V, as the physical trauma and the lymphocytic infiltration emerging. It is worth to mention that skin damage caused by needle-based electroporation was found at not only the needle-injection foci, but also the area between the two needles (lower right in figure (c)). During a 7-days period, the skin showed a recovery process, but obvious damage on skin could be found by both visual check and section analysis. In contrary, no pathological change was observed for the skins treated with ep-Patch at 70 V (middle left in figure (d)). However, in order to focus the electric field on a restricted area and avoid the unnecessary influence or safety risk on irrelevant tissues, the ep-Patch was designed to directly attach to the surface of the tissue of interest. Therefore the damage of the skin became worse while the voltage increased from 90 V to 130 V. In the worst-case scenario, the stratum corneum and the epidermis were destructed (lower left in figure (d)). Even though, for 70 ~ 110 V, the recovery processes were finished in a 7-days period, confirmed by both histological examination (such as the middle right image in figure (d)) and naked-eye observation of the skin appearance. For 130 V, even the structure of the skin was repaired, there were still some inflammation, hinting that a longer period was required for a full recovery.

Along with the histological examination, other experimental phenomena also suggested that ep-Patch was superior to needle-based device regarding to introduction of tissue damage. For example, when we collected specimens of mice treated with ep-Patch at indicated time points, the skin and muscle could be separated simply by peeling with blunt edges of scissors. While for needle-electroporated animal, since obvious pathological changes occurred, it is difficult to separate them by peeling, and direct cutting with sharp edges usually was required.

To conclude, needle-based electroporation caused severe damage on both muscle and skin tissues. In contrast, our ep-Patch was proved to be safe for muscle in a voltage range from 70 V to 130 V. Concerning the skin, the safe range of voltage should be limited below 90 V for ep-Patch, which was enough for non-invasive electroporation. In addition, the skin damaged caused by the ep-Patch was recovered within 7 days.

**Supplementary Figure S7**

**Flexible parylene films coated with unpatterned gold layer as electroporation electrodes**

**
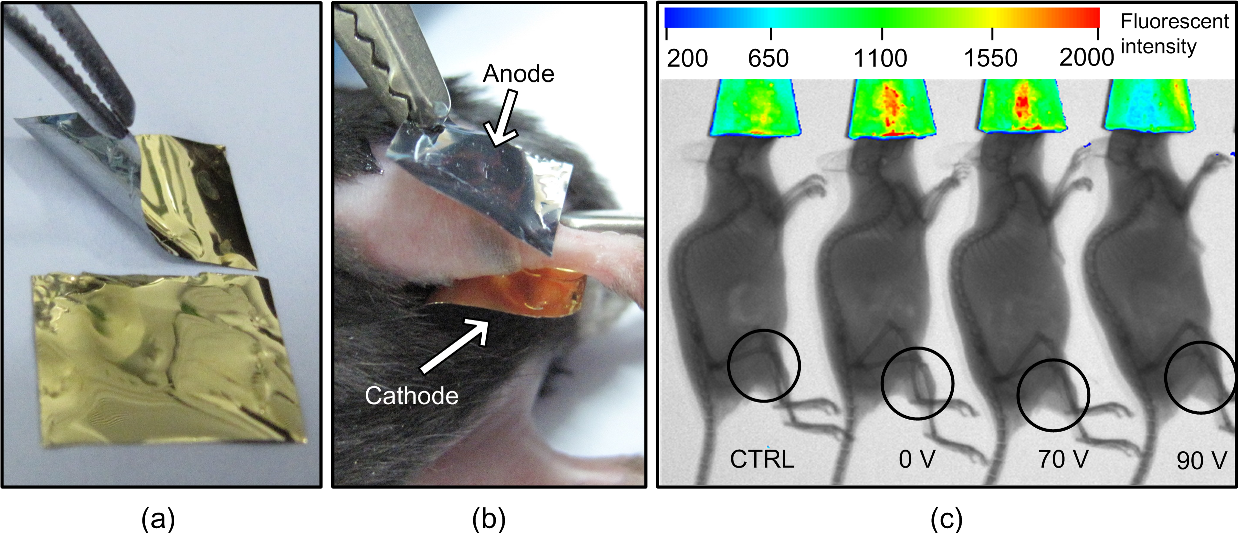
**

(a) Using the method described in Supplementary Figure S2a (skipped the patterning step), two flexible parylene films coated with intact gold layer were fabricated as electroporation electrodes. The thicknesses of the parylene film and the gold layer were 10 μm and 0.3 μm, respectively. Differed from using only one ep-Patch to cover the mouse leg, two parylene films were required as anode and cathode. To fit the size of mouse leg and avoid short circuit, both two parylene films were shrunk to 1 cm in length and 7 mm in width.

(b) While electroporating mouse leg, two parylene films were placed on the opposite sides of mouse leg as anode and cathode, respectively. To clearly demonstrate the configuration of two parylene films, both films were attached loosely. While processing electroporation, the electroporation procedure, electrical parameters and the amount of RFP plasmid were the same with that used in the non-invasive ep-Patch assays.

(c) The whole body fluorescence intensity imaging indicates no successful electrotransfection occurred with both 70 V and 90 V. The leftmost mouse was without any treatment, which serves as negative control. The other three mice were all injected with hyaluronidase and RFP plasmid, followed by electrical stimulation with 0 V, 70 V and 90 V, respectively. The skin was removed before imaging. The black circuits label the treatment area. No fluorescent protein expression was observed both 24 hours (not shown) and 48 hours (as shown in figure c) after electrical stimulation. As a contrast, 70 V is sufficient for electrotransfection while utilizing the ep-Patch.

**Supplementary Figure S8**

**The measurement of the current passing through the ep-Patch.**


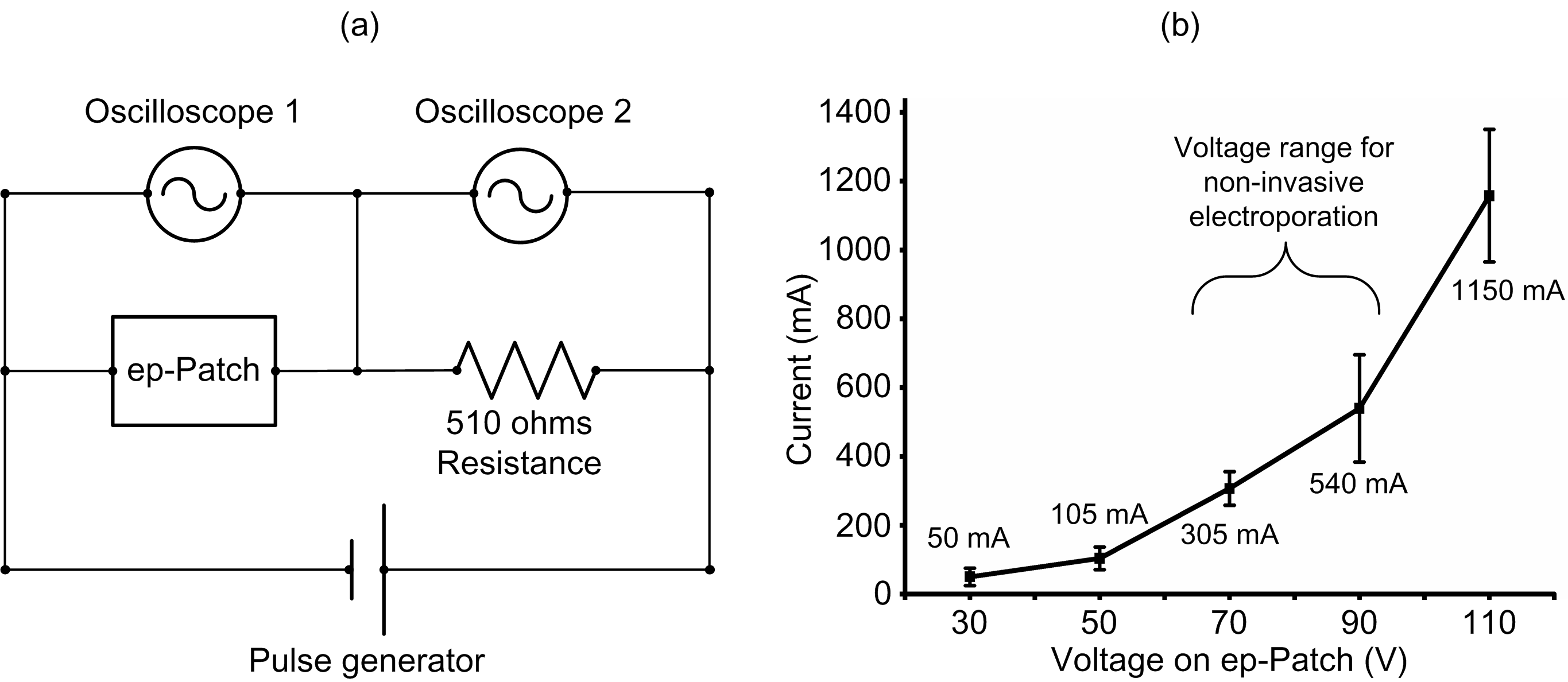


(a) The scheme of the measuring circuit:

The impedance of the skin was complicate, varying according to many factors, such as the physical status of skin, the loss of the stratum corneum, the amplitude and frequency of the applied voltage. Therefore, we cannot simply calculate the ep-Patch current by monitoring the voltage drop on chip. To acquire the current passing through the ep-Patch, a 510 ohms resistance was connected in series with the ep-Patch, and two oscilloscopes were employed to measure the voltage drops on ep-Patch and resistance, respectively. After attaching the ep-Patch on depilated mouse, the voltage drop on the ep-Patch was monitored through the oscilloscope 1. By varying the output value of the pulse generator, the voltage drop on ep-Patch could be adjusted to a required number (such as 70 V for non-invasive electroporation). At this moment, the voltage drop on the resistance could be read out on the oscilloscope 2. By dividing the voltage by the resistance, we calculated the current passing through the 510 ohms resistance. In this series circuit, the ep-Patch current equaled to the current on the resistance.

(b) The ep-Patch current under different voltages:

The current values under different voltages were acquired by three independent assays. Each bar represents the mean ± S.D. The thickness of the parylene film was 10 μm, and the thickness of the gold electrode was 12 μm. In the voltage range for non-invasive electroporation in our assays (70 ~ 90 V), the chip current was from 305 mA to 540 mA (as shown in figure (b)).

**References**

1. Huang H*, et al.* An efficient and high-throughput electroporation microchip applicable for siRNA delivery. *Lab Chip* **11**, 163-172 (2011).

2. Kooijmans SA*, et al.* Electroporation-induced siRNA precipitation obscures the efficiency of siRNA loading into extracellular vesicles. *J Control Release* **172**, 229-238 (2013).

3. Cutrera J, King G, Jones P, Gumpel E, Xia X, Li S. Managing local swelling following intratumoral electro-chemo-gene therapy. *Methods Mol Biol* **1121**, 233-239 (2014).

4. Gehl J. Gene electrotransfer in clinical trials. *Methods Mol Biol* **1121**, 241-246 (2014).

5. Spugnini EP*, et al.* Electroporation as a strategy to promote HtrA1 gene uptake and chemotherapy efficacy in a mouse model of mesothelioma. *Front Biosci (Elite Ed)* **5**, 974-981 (2013).

6. Albrecht MT, Livingston BD, Pesce JT, Bell MG, Hannaman D, Keane-Myers AM. Electroporation of a multivalent DNA vaccine cocktail elicits a protective immune response against anthrax and plague. *Vaccine* **30**, 4872-4883 (2012).

7. Chabot S, Rosazza C, Golzio M, Zumbusch A, Teissie J, Rols MP. Nucleic acids electro-transfer: from bench to bedside. *Curr Drug Metab* **14**, 300-308 (2013).
